# Behavioral Abnormalities in a Mouse Model of Chronic Toxoplasmosis Are Associated with MAG1 Antibody Levels and Cyst Burden

**DOI:** 10.1371/journal.pntd.0004674

**Published:** 2016-04-28

**Authors:** Jianchun Xiao, Ye Li, Emese Prandovszky, Geetha Kannan, Raphael P. Viscidi, Mikhail V. Pletnikov, Robert H. Yolken

**Affiliations:** 1 Stanley Division of Developmental Neurovirology, Department of Pediatrics, Johns Hopkins School of Medicine, Baltimore, Maryland, United States of America; 2 Department of Psychiatry, Johns Hopkins University School of Medicine, Baltimore, Maryland, United States of America; 3 Department of Pediatrics, Johns Hopkins School of Medicine, Baltimore, Maryland, United States of America; Hitit University, Faculty of Medicine, TURKEY

## Abstract

There is marked variation in the human response to *Toxoplasma gondii* infection. Epidemiological studies indicate associations between strain virulence and severity of toxoplasmosis. Animal studies on the pathogenic effect of chronic infection focused on relatively avirulent strains (e.g. type II) because they can easily establish latent infections in mice, defined by the presence of bradyzoite-containing cysts. To provide insight into virulent strain-related severity of human toxoplasmosis, we established a chronic model of the virulent type I strain using outbred mice. We found that type I-exposed mice displayed variable outcomes ranging from aborted to severe infections. According to antibody profiles, we found that most of mice generated antibodies against *T*. *gondii* organism but varied greatly in the production of antibodies against matrix antigen MAG1. There was a strong correlation between MAG1 antibody level and brain cyst burden in chronically infected mice (r = 0.82, p = 0.0021). We found that mice with high MAG1 antibody level displayed lower weight, behavioral changes, altered levels of gene expression and immune activation. The most striking change in behavior we discovered was a blunted response to amphetamine-trigged locomotor activity. The extent of most changes was directly correlated with levels of MAG1 antibody. These changes were not found in mice with less cyst burden or mice that were acutely but not chronically infected. Our finding highlights the critical role of cyst burden in a range of disease severity during chronic infection, the predictive value of MAG1 antibody level to brain cyst burden and to changes in behavior or other pathology in chronically infected mice. Our finding may have important implications for understanding the heterogeneous effects of *T*. *gondii* infections in human.

## Author Summary

Chronic infection with *Toxoplasma gondii*, a common neurotropic pathogen, affects approximately 1 billion people worldwide. There is marked variation in the human response to infection. Epidemiological studies indicate that virulent strains of *T*. *gondii* are associated with increased frequency and severity of human toxoplasmosis. However, animal studies on the pathogenic effect of chronic infection have focused on relatively avirulent strains because they can easily establish latent infections in mice. Employing a murine model of chronic infection with virulent strain, we found that mice displayed variable outcomes ranging from aborted to severe infections. Parasite burden, as measured serologically, was found to be the critical determinant of behavioral and pathogenic changes in chronically infected mice. This study demonstrated the predictive value of serologically determined parasite burden to the levels of chronic infection. Our finding highlights the critical role of parasite burden in a range of disease severity during chronic infection. Our finding may have important implications for understanding the heterogeneous effects of *T*. *gondii* infections in human.

## Introduction

The intracellular protozoan *Toxoplasma gondii* is an exceptionally successful parasite that infects approximately 1 billion people worldwide. Latent infection persists during the lifespan of the intermediate hosts such as human through the formation of cysts in muscle and brain. Although genotyping of *T*. *gondii* isolates from all continents reveals a complex population structure [1], the majority of strains isolated in North America and Europe fall into one of three clonal lineages: types I, II and III [2]. Among the three clonal lineages, type I strains are lethal in mice, but type II and III strains are considerably less virulent. Pathogenicity differences among the three strains are largely determined by genetic polymorphisms and differences in expression level of secretory proteins released from dense granule and rhoptry organelles (e.g. GRA15, ROP5, ROP16 and ROP18) [3].

Human infections with *T*. *gondii* display a wide range of clinical symptoms. This variation is likely to be a consequence of many factors including human and parasite genotypes, timing of infection, and environmental factors such as co-infections and nutrition. Regarding parasite genotypes, virulent strains are found to be associated with increased frequency and severity of human toxoplasmosis [4]. For example, several studies have suggested that type I strains are more pathogenic in immunocompromised patients. Khan et al. [5] analyzed 11 cerebral spinal fluid (CSF) samples collected from patients who had confirmed or presumptive toxoplasmosis encephalitis. They found a majority of these patients had infections with type I strains or strains containing type I alleles. Ferreira et al. [6] investigated the genotypes of *T*. *gondii* strains isolated from 87 patients with cerebral toxoplasmosis and AIDS, treated in Sao Paulo State, Brazil. Although their study revealed a high rate of genetic polymorphism in *T*. *gondii* strains, type I seems to be most prevalent as this strain was responsible for infection in 46% of their patients. In a small series of patients with severe ocular inflammation from the United States, there was an unusual abundance of type I or atypical parasites [7]. Evidence from serotyping indicated that the offspring of mothers with *T*. *gondii* type I infection were at significantly increased risk for the development of psychoses as compared with the matched unaffected control mothers [8]. In the case of congenital toxoplasmosis, McLeod et al. [9] determined the parasite serotype for 193 congenitally infected infants and their mothers in the NCCCTS, 1981–2009. The authors noted that NE-II (all non-type II) serotypes are more often present than type II serotypes in infants with congenital toxoplasmosis (61% vs. 39%) in the United States. Moreover, infants with NE-II serotypes are more likely to experience severe disease at birth than those with type II serotypes. Several hypotheses such as strain-related differences in pathogenicity, poor host (human) adaption and human genetic predisposition have been suggested to account for the strain-specific pathology [10–12].

Type I strains of *T*. *gondii* are excellent *in vitro* models, but most strains of this type do not readily develop tissue cysts or latent infection in laboratory mice. In contrast, type II strains easily establish latent infections in mice characterized by the presence of tissue cysts, a fact that could explain their common usage for chronic studies. Given the association of type I strains with severity of human toxoplasmosis [4], we sought to investigate its chronic effect using a murine model. The effect of type I strain infection was assessed by changes on behavior, cytokine and gene expression. Previously, we have developed an assay which measures humoral immune response against *T*. *gondii* matrix antigen MAG1 using synthetic peptides. Employing samples from patients with and without clinical toxoplasmosis, we have shown that the MAG1 antibody level can differentiate active from inactive human toxoplasmosis [13]. We thus hypothesized that behavioral change or the presence of pathology in chronically infected mice may be associated with high levels of MAG1 antibody.

## Materials and Methods

### Mouse model of chronic *T*. *gondii* type I infection

Seven- to nine-week-old female outbred CD-1 mice (ICR-Harlan Sprague) were infected (n = 46) intraperitoneally with 500 tachyzoites of *T*. *gondii* GT1 strain (type I, virulent) diluted in 200 μL of phosphate-buffered saline (PBS). For controls, un-infected mice received 200 μL of only the vehicle (PBS) intraperitoneally (n = 18). To establish a chronic infection, both control and infected mice were treated with anti-*T*. *gondii* chemotherapy (sulfadiazine sodium) in drinking water (400 mg/L, Sigma) from day 5 to day 30 post-infection. This treatment is to control the proliferation of tachyzoites during the acute stage and to avoid animal death. Sulfadiazine is a competitive analogue of PABA and inhibits tachyzoite growth, but not encysted bradyzoites [14]. The GT1 strain was maintained by passage in human fibroblast cells (HFF, ATCC SCRC-1041).

### Ethics statement

All protocols (#MO15M17) were approved by the Animal Care and Use Committee at Johns Hopkins University. All experiments conformed to the U.S. National Institutes of Health Guide for the Care and Use of Laboratory Animals.

### MAG1 antibody generation profile in type I-infected mice over time

To determine the kinetics of MAG1 antibody generation in mice infected with the GT1 strain, sera samples from a pilot mouse cohort (n = 10) were collected weekly from the tail vein between weeks 2 and 18 following infection. The development of MAG1 antibody was monitored using the MAG1 ELISA assay, which measures antibodies directed against cyst antigen MAG1 using synthetic peptides (MAG1_4 and MAG1_5) [13]. The MAG1 antibody level was reported from either MAG1-4 or MAG1-5 depending on whichever one was higher. *T*. *gondii* infection was also confirmed by measuring anti-*T*. *gondii* IgG using a commercial ELISA assay (VIR-ELISA, Viro-Immun Labor-Diagnostika, Oberursel Germany) modified as previously described [8]. The primary antibody consisted of diluted serum (1:100) and secondary antibody was enzyme labeled anti-mouse IgG.

### Behavioral assays

Mice were subjected to behavioral assessments between 12 and 21 weeks postinfection (wpi), at the time when mice are expected to have their maximum levels of MAG1 antibody. The tests were conducted in the following order: novelty-induced activity in the open-field, spatial working and recognition memory in the Y-maze, novel object recognition, and amphetamine-induced activity in the open field.

### Novelty-induced activity

Novelty-induced activity was examined using activity chambers with infrared beams (San Diego Instruments Inc.), as previously described [15]. General locomotor activity was recorded as the number of beams broken and exploration was assessed as the number of rears for a 30-min period.

### Spatial working and recognition memory

As previously described [16], the test included two sessions to evaluate spatial working and recognition memory, respectively, in a Y-maze. In the first session we scored the number of alternations done by the mouse when all three arms were visited, without entering the same arm twice in a row. After 5 days, the second session was performed and consisted of two trials. During trial 1, one arm of the maze was blocked and a mouse was allowed to freely explore the two open arms for 5 min. After a 20-min delay, trial 2 began during which the block was removed and the mouse was allowed to freely explore all three open arms for 5 min. The percentage of time and visits into the novel (previously blocked) arm during the first 2 min of the 5-min trial was analyzed [17].

### Novel object recognition

As previously described [18], mice were habituated for 3 days to an empty cage for 10 min each day. On day 4, each mouse was subjected to three successive (habituation, pretest and test) sessions. After each session, there was a retention interval of 1 h, during which the mouse was held in its home cage. During the pretest session, two identical objects (object A) were placed on opposite ends of the empty cage, and the mouse was allowed to freely explore the objects for 10 min. During the test session, one of the two familiar objects was replaced with a novel one (object B), and the mouse was allowed to freely explore the familiar and novel object for 5 min. After 24 h, mouse was returned to the empty cage, which now contained an entirely novel object, C, and one of the familiar objects, A, and was tested for a duration of 5 min. The exploratory preference was calculated as the time near the novel object divided by the total time near either object. All the test objects have been extensively validated previously to ensure that no intrinsic preferences or aversions exist and that the animals explore all the objects for similar durations.

### Amphetamine-induced activity

Each mouse was subjected to three successive (habituation, saline injection and amphetamine injection) sessions. Briefly, mice were first placed into an open-field chamber (San Diego Instruments Inc.) with their baseline activity recorded for 30 min, then injected intraperitoneally with 0.9% saline with their locomotor activity recorded for 30 additional min. Finally, mice were injected with D-Amphetamine sulfate (Sigma, St. Louis, MO) at doses of 2.5 mg/kg body weight and recorded for a final 60 min.

### Collection of mouse tissue

Mice that completed the behavioral testing were sacrificed between 22 and 24 wpi by cervical dislocation and then decapitation. The whole brain was removed and the prefrontal cortex and striata were rapidly dissected on ice and stored at −80°C for subsequent experiments. For neurochemical analysis, one side of the striatum was snap frozen in liquid nitrogen, and stored at −80°C. Upon sacrifice, blood samples were collected and serum was isolated.

### Quantitative PCR

Total RNA was extracted using the miRNEasy kit (Qiagen). Reverse transcription was performed using either Multiscribe reverse transcriptase and random primers (Applied Biosystems, Foster City, CA, USA) to generate cDNA, or the Multiscribe miRNA Reverse Transcription kit (Applied Biosystems) using miRNA-specific primers to produce miRNA. Quantitative PCR was performed using inventoried miRNA assays (Applied Biosystems) with standard ABI protocols and reagents, as previously described [19]. The fold changes between groups were evaluated using relative quantization (delta Ct method) with b-actin as an endogenous mRNA control and RNU48 as an endogenous miRNA control. All the qPCR analyses were repeated at least three times to confirm differences in the expression levels and only results consistent across all three analyses were considered valid. For the mouse striatum, gene expression of miR-132 was measured. For the mouse cortex, expressions of BAG1 [20] and SAG1 [21] were measured.

### Measurement of dopaminergic and serotoninergic amines by HPLC

Biogenic amine concentrations of striatal tissues were measured by high performance liquid chromatography with electrochemical detection (HPLC-ECD), as previously described [19]. Data were normalized to protein concentrations (ng neurotransmitters/μg protein).

### Bio-Plex protein expression assay

The levels of 23 different cytokines and chemokines were measured in the prefrontal cortex of the mouse brain using Bio-Plex multiplex assay (Bio-Rad) according to the manufacturer’s instructions.

### Cyst counts

In a separate cohort of the same infection model, mice were sacrificed at 20 wpi and brains were removed and cut sagitally along the midline. For each mouse, half of the brain was homogenized in 400 μl of PBS containing 0.2% Triton X-100. A 1:2000 dilution of DAPI and a 1:200 dilution of fluorescein Dolichos biflorus agglutinin were added to an aliquot of the brain suspension and incubated on ice for 5 minutes. The aliquot was examined using a fluorescent microscope, and the number of brain cysts was determined in six (for mice with high MAG1level) or twelve (for mice with low MAG1 or without MAG1 level) samples of 8 μl suspension per each brain homogenate at 400X magnification. All numbers reported correspond to the numbers obtained for the half-brain multiplied by 2 for comparison with published data for the whole brain. The size of the cyst was determined using the maximum diameter of the cyst.

### Statistical analyses

Data are presented as means ± SEM. Based on the antibody profiles, mice were divided into five groups for analysis: control, mice with MAG1 high (IgG+/MAG1+high), mice with MAG1 low (IgG+/MAG1+low), mice without MAG1 (IgG+/MAG1-), and mice being exposed but didn’t develop any antibodies (IgG-/MAG1-). Initial analysis showed that results were normally distributed. Generally, behavior and qPCR data were analyzed by one-way ANOVA. For monitoring body weight and amphetamine-induced activity, a repeated measures ANOVA was applied with group as the between-subject factor and time as the within-subjects factor. Further post hoc Bonferroni testing was conducted to explore any significant main effects resulting from the ANOVAs. Correlation analysis between MAG1 antibody level and measured parameters was performed using two-tailed Spearman’s correlation coefficient (r). The correlation analysis only involved samples which are considered MAG1 seropositive. The effect of cytokine on behavior responses was examined by logistic regression analysis with and without adjusting for serological status (*T*. *gondii* and MAG1 antibody). Variables included in the regression models were selected based on known risk factors for *T*. *gondii* infection.

Statistical analyses were conducted in SPSS (Version 21.0, SPSS, Chicago, IL, USA), GraphPad Prism V5.02 (GraphPad Software Inc., La Jolla, CA, USA) and STATA version 12 (STATA Corp LP, College Station, Texas, U.S.A.). Significance was denoted as p < 0.05.

## Results

### Kinetics of antibody response to *T*. *gondii* MAG1 antigen

**Fig 1:**
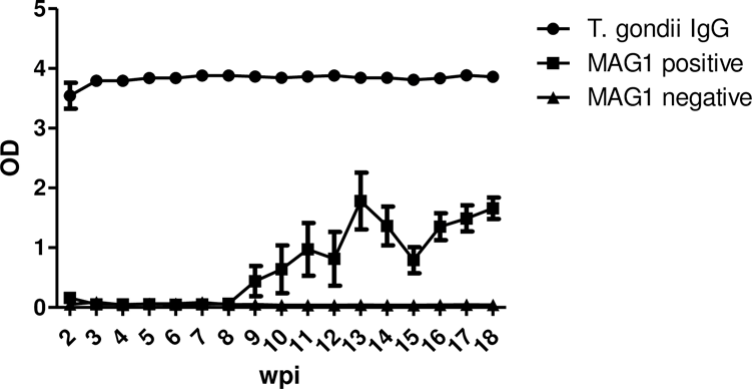
Kinetics of the humoral response against *T*. *gondii* organism and MAG1 peptides following *T*. *gondii* type I infection. Female CD-1 mice (7–9 weeks old, n = 10) were infected intraperitoneally with 500 tachyzoites of GT1 strain and sera samples were collected weekly from 2 to 18 weeks after infection. About half of these mice (MAG1 positive, n = 5) developed MAG1 antibody response, while the other half (MAG1 negative, n = 5) failed to generate MAG1 antibody. Levels of *T*. *gondii* IgG and MAG1 antibody were determined as described in materials and methods. Error bars denote SEM.

Because GT1 is a virulent strain, we administered anti-*T*. *gondii* chemotherapy to ensure mouse survival. To investigate the kinetics of antibody response, a pilot study (n = 10) with sera collected weekly from 2 to 18 weeks after infection was examined. We measured antibody response to both *T*. *gondii* organism and MAG1 peptides (MAG1_4 and MAG1_5). As seen in Fig 1, anti-*T*. *gondii* IgG antibodies were detected at 2 wpi, peaked at 3 wpi, and remained high thereafter, in all GT1-exposed mice. However, only half of these mice (n = 5) developed MAG1 antibody response, referred to MAG1 positive. The other half failed to generate MAG1 antibody (MAG1 negative, n = 5). MAG1 antibody seroconversion occurred at variable time points between 3 to 11 wpi, then reached a peak response at approximately 13 wpi and remained relatively stable thereafter. Neither *T*. *gondii* nor MAG1 antibodies were present in the control group (n = 3, data not shown).

### Characterization of type I-exposed mice

Since our pilot study suggested that GT1-exposed mice displayed variability in the MAG1 antibody generation, we first characterized antibody profiles in mice used for behavioral assessment. After the last behavioral test, sera were collected and measured for their antibody levels to *T*. *gondii* and MAG1 antigen. For *T*. *gondii* IgG antibody, 72% of the GT1-exposed mice (33 out of 46) were seropositive and there was little inter-animal variation in the antibody levels (IgG = 3.72 ± 0.09, mean ± SEM). For MAG1 antibody, the cut-off value for a positive response (OD = 0.07) was defined as mean plus 4 standard deviation of the control samples. Using this value, MAG1 antibody was detected in 46% of GT1-exposed mice (21 out of 46) and their levels varied greatly (MAG1 = 1.23 ± 0.30). Therefore, mice were stratified into high MAG1 level (MAG1 = 2.38 ± 0.36, n = 10) and low MAG1 level (MAG1 = 0.19 ± 0.04, n = 11) groups based on the distribution of MAG1 antibody levels. According to antibody profiles, we categorized the mice into 5 groups, as follows: (i) unexposed control (contain neither of these antibodies, n = 18); (ii) mice with high MAG1 antibody level (IgG+/MAG1+high, MAG1 antibody level > 0.5, n = 10); (iii) mice with low MAG1 antibody level (IgG+/MAG1+low, MAG1 antibody level < 0.5, n = 11); (iv) mice without MAG1 antibody (IgG+/MAG1-, n = 12); (v) mice exposed to *T*. *gondii* that did not develop any antibody response (IgG-/MAG1-, n = 13).

Because MAG1 antigen is abundantly expressed in the cyst matrix, we next investigated the relationship between MAG1 antibody level and the number of tissue cysts in the brain (Table 1). In a separate cohort of mice 20 wpi, we measured the number of tissue cysts in half of the brain in *T*. *gondii*-seropositive mice (IgG+, n = 16). Tissue cysts of *T*. *gondii* were found in brains of all MAG1-seropositive mice (MAG1+, n = 11), while no cyst was detected in MAG1-seronegative mice (MAG1-, n = 5). Using Spearman Rank analysis, significant positive correlation between MAG1 antibody level and the number of brain cysts was found (r = 0.82, p = 0.0021, Table 1). Moreover, there was also a significant correlation between MAG1 antibody level and the average size of tissue cysts within the brain (r = 0.89, p = 0.0003, Table 1). If mice were categorized into the predefined MAG1+high or MAG1+low group, there was a significant difference in cyst burden between the groups (median cyst burden for MAG1+high, 175.5, and MAG1+low, 52.0, p = 0.0043, Mann-Whitney test)

**Table 1:** Quantitative data on the number and size of brain tissue cysts, and *T*. *gondii* MAG1 antibody levels.

MAG1 antibody level	Cyst No./per brain	Average cyst size ± SEM (μm)
3.681	164	37 ± 6.19
2.966	201	25 ± 10.70
2.804	236	25 ± 2.33
2.377	187	22 ± 3.04
1.128	97	12 ± 1.08
0.938	82	15 ± 4.60
0.486	52	10 ± 0.85
0.339	41	8 ± 1.23
0.283	58	7 ± 1.27
0.220	43	11 ± 0.78
0.197	56	9 ± 0.90
	r = 0.82^a^	r = 0.89^b^
	p = 0.0021^a^	p = 0.0003^b^

^a^Spearman’s correlation coefficient (r) and p value between MAG1 antibody level and the number of brain tissue cysts.

^b^Spearman’s correlation coefficient (r) and p value between MAG1 antibody level and the average size of brain tissue cysts.

Furthermore, the relationship between MAG1 antibody level and expression of bradyzoite-specific gene (BAG1) was investigated in the prefrontal cortex of mouse. The expression of BAG1 gene was detected by qPCR and sample was considered positive after reaching the threshold (< 40 cycles). Our results showed that the positive rate of BAG1 expression was associated with MAG1 antibody level (p = 0.011). In mice with high MAG1 levels, the positivity rate was 87.5% (7 out of 8). In contrast, the positivity rate was lower in mice with low MAG1 levels (25%, 2 out of 8). No BAG1 gene expression was detected in unexposed control mice, exposed mice without IgG antibody (IgG-/MAG1-), or mice without MAG1 antibody (IgG+/MAG1-). To identify whether there is bradyzoite-to-tachyzoite interconversion, we measured the expression of tachyzoite specific gene SAG1 among different groups of mice. Our results demonstrated that SAG1 expression was undetectable in samples from all the GT1-exposed mice (data not shown).

### Poor body weight gain in mice with high MAG1 level

**Fig 2:**
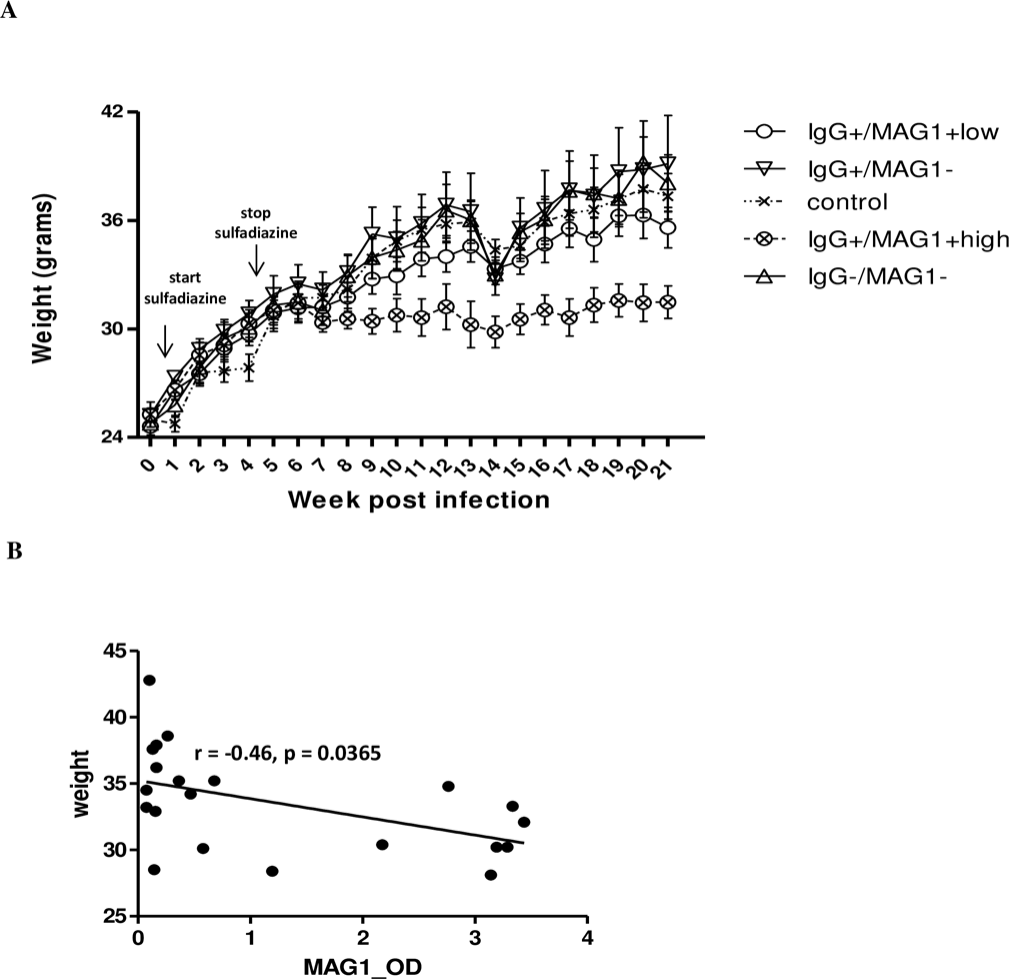
(A) Mice with high MAG1 antibody level exhibited lower weight over the course of *T*. *gondii* infection. Female CD-1 mice (n = 46) were infected intraperitoneally with 500 tachyzoites of GT1 strain at 7–9 weeks of age. Mice were weighed weekly and weights were analyzed according to their antibody profiles (*T*. *gondii* and MAG1 antibody) by repeated measures ANOVA. (B) Levels of MAG1 antibody inversely correlated with body weight of mice (Spearman’s correlation analysis). Error bars denote SEM.

To examine the effect of chronic *T*. *gondii* GT1 infection on body weight, mice were weighed weekly and weights were analyzed by repeated measures ANOVA with group as a between-subject factor and time as a within-subjects factor. The results revealed a significant effect for time, F (4,59) = 51.303, p < 0.0005, and a significant interaction between time and group, F (4, 59) = 1.642, p = 0.004 (Wilks’ Lambda). As seen in Fig 2A, all groups of mice showed a weight gain before week 6 post infection. Although other groups of mice continued to have weight gain throughout the experiment, the group of mice with high MAG1 antibody level displayed weight loss at 7 wpi (e.g. 2 weeks after stopping sulfadiazine treatment) and has poor weight gain since then.

We observed a negative correlation (r = -0.46, p = 0.0365, Fig 2B) between levels of MAG1 antibody and body weight. We also examined if mouse starting weight had an impact on generation of MAG1 antibody, but no significant correlation was found (p = 0.5925).

### Behavioral deficits in mice with high MAG1 level

Behavioral testing was conducted between 12 to 21 weeks following infection. In summary, mice with high MAG1 antibody level exhibited reduced locomotor and exploratory activity, impaired object recognition memory when tested at a 24 h delay, and lack of response to amphetamine induced activity. No significant group differences occurred in the Y-maze test which was used to evaluate spatial working and recognition memory (Ps > 0.5).

**Fig 3:**
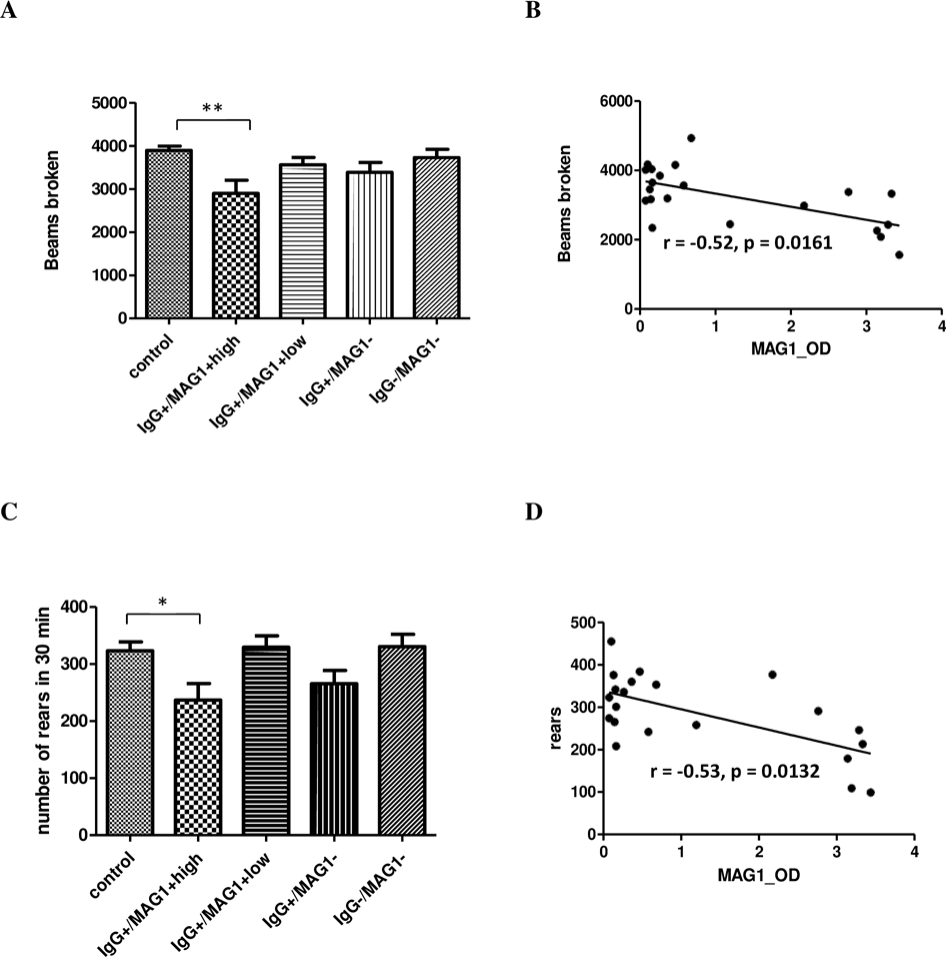
Open field behavior in a 30 min test period indicated that mice with high MAG1 level have lower levels of (A) general locomotor activity and (C) reduced rearing, as compared to the controls. MAG1 antibody level inversely correlates with (B) locomotor activity and (D) rears (Spearman’s correlation analysis). ANOVA followed by Bonferroni’s post hoc: *p < 0.05, **p < 0.01. Error bars denote SEM.

As a commonly used model to assess novelty-induced activity, we conducted open field test to evaluate general locomotor and exploratory activity in *T*. *gondii* GT1-infected mice. As depicted in Fig 3A, mice with high MAG1 antibody level showed decreased locomotor activity, as evidenced by the significantly fewer number of broken beams compared to control (F(4, 59) = 3.803, p = 0.0081; post Bonferroni’s test control vs IgG+/MAG1+high, p < 0.01). Similarly, exploration, assessed by the number of rears, was also significantly reduced in the group of mice with high MAG1 level (F(4, 59) = 4.609, p = 0.0026; post-hoc Bonferroni’s test control vs IgG+/MAG1+high, p < 0.05, Fig 3C). Both locomotor activity and rearing were negatively correlated with levels of MAG1 antibody (r = -0.52, p = 0.0161, r = -0.53, p = 0.0132, respectively, Fig 3B and 3D).

**Fig 4:**
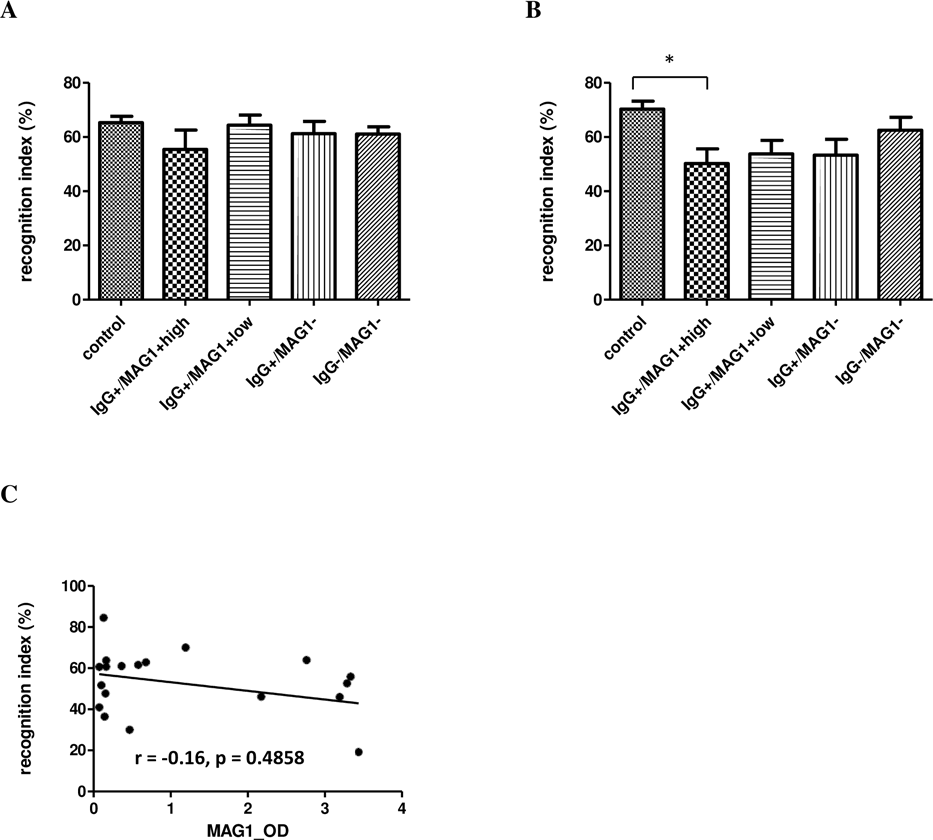
Object recognition memory was impaired in mice with high MAG1 level when tested at a 24 hour delay (B), but not at a 1 h delay (A). (C) MAG1 antibody level and cognitive performance were not significantly correlated (Spearman’s correlation analysis). ANOVA followed by Bonferroni’s post hoc: *p < 0.05 compared with controls. Error bars denote SEM.

The effect of *T*. *gondii* infection on recognition memory of mice was assessed by novel object recognition, which utilizes the innate tendency of rodents to preferentially explore novel objects. A preference score of 50% indicates random exploration, while scores higher than chance reflect intact memory. As seen in Fig 4A, all groups of GT1-exposed mice had normal recognition memory after a retention interval of 1 h (F(4, 59) = 0.9017, p = 0.4689, Fig 4A). However, after a retention interval of 24 h, all *T*. *gondii* IgG seropositive mice (IgG+) failed to show a preference for the novel object, as evidenced by they had lower recognition indexes compared to control (mice with high MAG1antibody, 50.2%; mice with low MAG1antibody, 53.7%; mice without MAG1antibody, 53.2%; control, 70.3%, Fig 4B). The difference between control and mice with high MAG1 antibody level was significant (F(4, 57) = 3.676, p = 0.0099; post-hoc Bonferroni’s test p < 0.05), but did not reach significance in mice with MAG1 low and without MAG1 groups. There was no correlation between MAG1 antibody level and cognitive performance at 24 h delay (r = -0.116, p = 0.4858, Fig 4C).

**Fig 5:**
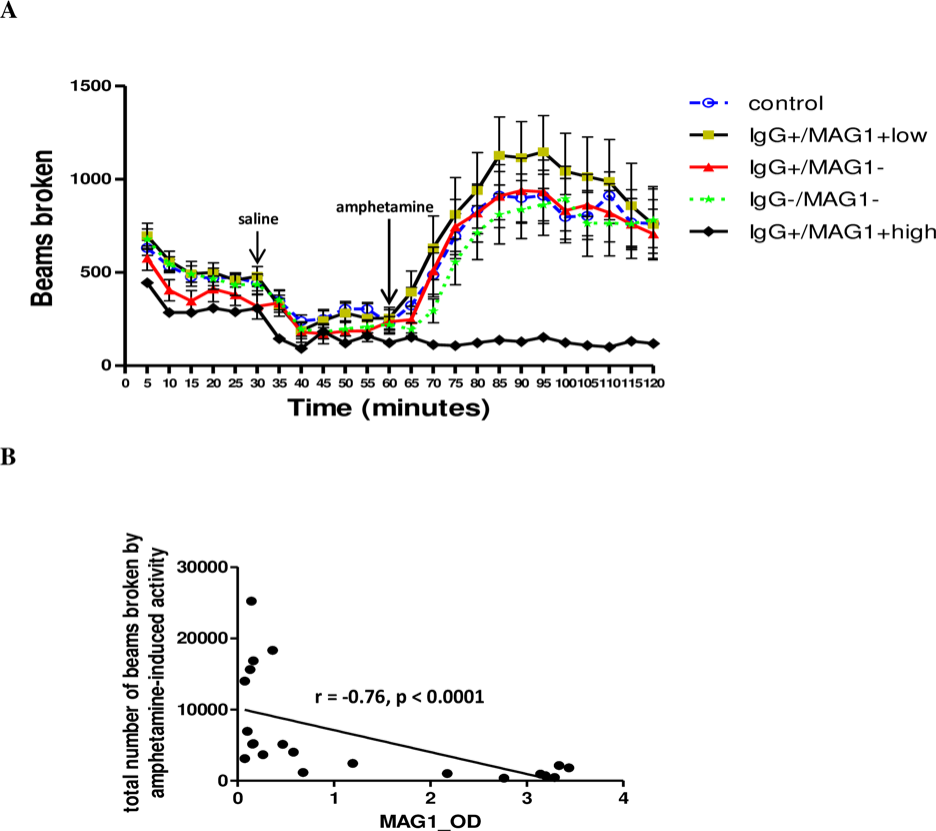
(A) Mice with high MAG1 level showed no response to amphetamine-triggered activity. Mice were subjected to three successive (habituation, saline injection and amphetamine injection) sessions with their locomotor activity recorded throughout. D-Amphetamine sulfate: 2.5 mg/kg body weight. (B) MAG1 antibody level inversely correlates with amphetamine-induced locomotor activity (Spearman’s correlation analysis). Error bars denote SEM. For the MAG1+high group the range is too low to be visualized (range of SEM, 3.78 to 15.34).

The role of dopamine has been suspected in *T*. *gondii*-induced behavioral alterations [22,23]. As a dopamine stimulant, we measured mouse response to amphetamine-trigged locomotor activity in the open field. As depicted in Fig 5A, mice with high MAG1 antibody level demonstrated a blunted response to amphetamine (one-way repeated measure ANOVA, F (4,59) = 6.562, p < 0.0005; post hoc Bonferroni Ps < 0.01 versus all other groups). In mice with low MAG1 level, the mean of amphetamine-trigged locomotor activity was higher than the other groups, but this was driven by a few mice displaying higher levels of activity and the difference was not significant (data not shown). Moreover, MAG1 antibody levels were significantly and inversely correlated with the total number of beams broken induced by amphetamine-triggered activity within the 60 min period (r = -0.76, p < 0.0001, Fig 5B).

### Brain cytokine profile

To examine inflammatory response to chronic *T*. *gondii* GT1 infection, we assessed 23 cytokine levels in the prefrontal cortex of mice from different groups. In addition, we examined relationships between these cytokine responses and behavior changes observed, with the intent to investigate whether inflammation related to these behavior responses.

**Fig 6:**
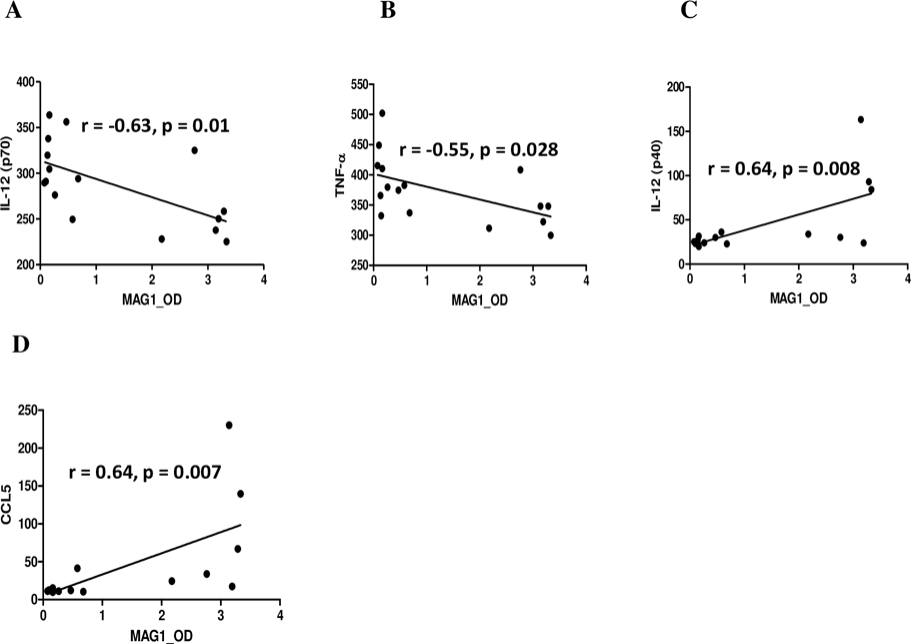
Correlations between brain cytokine concentrations and MAG1 antibody level. Five out of 23 cytokines differed significantly in concentrations between mice with high MAG1 levels and controls (ANOVA and Bonferroni correction). Spearman's correlation analysis was used to evaluate the relationship between the 5 cytokines and MAG1 antibody level. The MAG1 antibody levels inversely correlated with levels of (A) IL-12p70 and (B) TNF-α, but positively correlated with (C) IL-12(p40) and (D) CCL5.

Among the 23 cytokines, five differed significantly between controls and mice with high MAG1 antibody level (Table 2). In mice with high MAG1 levels, IL-1α (p = 0.003), IL-12p70 (p = 0.004), and TNF-α (p = 0.013) levels were decreased, while CCL5 (p = 0.008) and subunit IL-12p40 (p = 0.013) levels were elevated. We found that all altered cytokines except IL-1α displayed correlations with MAG1 antibody level. As depicted in Fig 6, MAG1 antibody level inversely correlated with levels of IL-12p70 and TNF-α (r = -0.63, p = 0.01; r = 0.-55, p = 0.028, respectively), but positively correlated with IL-12p40 and CCL5 (r = 0.64, p = 0.008; r = 0.64, p = 0.007, respectively).

**Table 2:** The concentration of significantly altered cytokines.

Group	Average concentration of cytokine/chemokine (pg/ml) ± SEM				
	IL-1α	IL-12p70	TNF-α	CCL5	IL-12p40
unexposed control	25.03 ± 1.32	367.99 ± 32.34	689.43 ± 150.83	15.88 ± 1.84	23.98 ± 1.36
IgG+/MAG1+high^a^	20.32 ± 0.52	258.54 ± 12.18	344.77 ± 12.78	70.55 ± 27.08	60.98 ± 17.52
IgG+/MAG1+low^b^	21.65 ± 1.13	317.42 ± 11.50	403.64 ± 18.85	12.16 ± 0.55	25.89 ± 1.33
IgG+/MAG1-^c^	23.67 ± 1.17	294.88 ± 15.55	377.51 ± 17.66	12.04 ± 1.27	22.81 ± 1.50
IgG-/MAG1-^d^	21.16 ± 0.61	344.25 ± 17.11	452.44 ± 30.45	11.86 ± 0.53	30.08 ± 1.14
p value^e^	**<**0.05	<0.01	**<**0.05	<0.05	<0.05

^a^IgG+/MAG1+high: mice with high MAG1 antibody level

^b^IgG+/MAG1+low: mice with low antibody MAG1 level

^c^IgG+/MAG1-: mice without MAG1 antibody

^d^IgG-/MAG1-: mice exposed to *T*. *gondii* that did not develop any antibody response.

^e^p value between IgG+/MAG1+high and unexposed control (ANOVA followed by Bonferroni’s post hoc).

N = 8 per group.

We then investigated whether these cytokine levels are related to behavioral changes. Logistic regressions, with or without adjusting for serological status (*T*. *gondii* and MAG1 antibody), were performed for all of the cytokines in relation to any of the 4 behaviors (novel activity, rearing, novel object recognition, and amphetamine-induced activity). The results of the unadjusted and adjusted cytokine analyses are shown in Table 3. In the adjusted analyses, we found that all cytokines except IL-1β were no longer significantly associated with behavioral responses. The adjusted p value for IL-1β was 0.042.

**Table 3:** Unadjusted and adjusted p values comparing behavior responses and cytokine levels^a^.

Behavior	Cytokine	Unadjusted p value	Adjusted p value
rearing	IL-1α	0.047	0.660
rearing	TNF-α	0.021	0.757
novel activity	IL-1α	0.029	0.728
novel object recognition	IL-6	0.014	0.181
novel object recognition	IL12p70	0.030	0.255
novel object recognition	TNF-α	0.027	0.146
amphetamine-induced activity	IL-1β	0.049	**0.042**
amphetamine-induced activity	IL-9	0.034	0.078
amphetamine-induced activity	IL-13	0.025	0.063
amphetamine-induced activity	Eotaxin	0.019	0.154

^a^The levels of cytokines were tested for association with behavior using a logistic regression model employing *T*. *gondii* and MAG1 antibody groups as covariates. Shown are regressions with R^2^ values above 0.10 (which corresponds to a p value ≤ 0.05) for all of the cytokines correlated with any of the 4 behaviors tested (novel activity, rearing, novel object recognition, and amphetamine-induced activity). After adjusting for serological status, all cytokines except IL-1β were no longer associated with behavior responses. The significant adjusted p value is indicated in bold.

### Neurotransmitter concentrations

Because changes in neurotransmitter concentration would affect behavioral performance, we measured biogenic amines and their metabolites in the mouse striatum. There were no significant differences (p > 0.05) in neurotransmitter (dopamine; serotonin) or metabolite (3,4-dihydroxyphenylacetic acid; homovanillic acid; 3-methoxy-4-hydroxyphenylglycol; and 5-hydroxyindoleacetic acid) concentrations among the five groups (data not shown).

### Gene expression of miR-132

**Fig 7:**
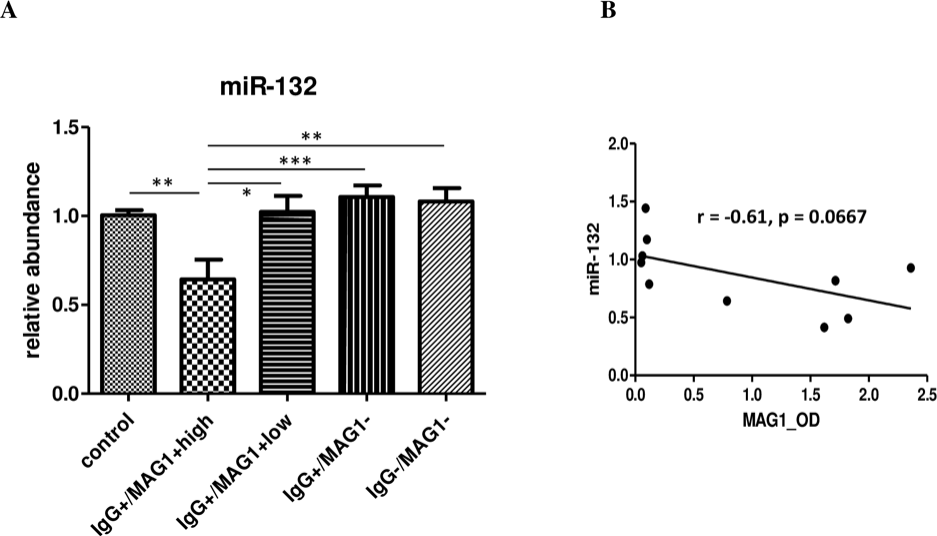
(A) miR-132 expression was decreased in mice with high MAG1 levels by qPCR analysis. (B) MAG1 antibody level showed a trend towards negative correlation with miR-132 expression (Spearman’s correlation analysis). ANOVA followed by Bonferroni’s post hoc: *p < 0.05, **p < 0.01, ***p < 0.001. Error bars denote SEM.

Because miR-132 alteration has been characterized in mouse models infected by *T*. *gondii* and its dysregulation has important implications for behavioral changes [19,24,25], we evaluated striatal miR-132 expression among different groups of mice. The selection of striatum is based on previous finding where significant change of miR-132 was noticed [19,24]. Employing qPCR analysis, we found a decrease in the expression of miR-132 in mice with high MAG1 level (F(4,28) = 6.403, p = 0.0009; Ps < 0.05 between IgG+/MAG1+high vs all other groups, Fig 7A). The expression of miR-132 showed a trend towards a negative correlation with MAG1 antibody level (r = -0.61, p = 0.0667, Fig 7B)

## Discussion

To provide insights into virulent strain-related severity of human toxoplasmosis, we established a chronic model of the virulent type I strain using outbred mice. We found that type I-exposed mice displayed variable outcomes ranging from aborted to severe infections, characterized by antibody profiles, pathology and behavior changes. The mechanisms by which infections either resolve in the acute phase or become chronic are not clear. However, differences in host immune response or sensitivity to anti-*T*. *gondii* chemotherapy might be involved. According to antibody profiles, we found that most of mice generated antibodies against *T*. *gondii* organism but varied greatly in the MAG1 antibody development. There was a strong correlation between MAG1 antibody level and brain cyst burden. We found that mice with high MAG1 antibody level displayed weight loss, behavioral changes, altered levels of gene expression and immune activation. The extent of most changes was directly correlated with levels of MAG1 antibody. These changes were not found in mice with less cyst burden or mice that were acutely but not chronically infected. Our finding highlights the critical role of cyst burden in a range of disease severity during chronic infection, the predictive value of MAG1 antibody level to brain cyst burden and to changes in behavior or other pathology in chronically infected mice.

In the present study, mouse response to infection was characterized by serum antibody levels to *T*. *gondii* organism and MAG1 antigen. Although it has been shown that MAG1 antigen is expressed during both tachyzoite and bradyzoite development [26], we found MAG1 antibody level was highly associated with cyst burden within the chronically infected brain, determined by the number of tissue cysts, by the average size of cysts, and by the bradyzoite prevalence in the mouse prefrontal cortex. Presumably, the association is due to the fact that MAG1 is abundantly expressed within the cyst and in the cyst wall surrounding the bradyzoites [26]. These results suggest that MAG1 antibody level is a parameter with predictive value with regard to cyst burden in infected mice.

We previously reported that MAG1 antibody level can distinguish active from inactive human toxoplasmosis [13]. Similarly, in the current study, the degree of symptomatology of brain pathology and behavioral changes in chronically infected mice was found to be associated with MAG1 antibody level. In contrast, many of these changes were not found in mice with low MAG1 antibody level or mice that lack of MAG1 antibody. Given MAG1 antibody level and brain cyst burden were highly correlated, this finding is in agreement with recent human studies reporting that the severity of toxoplasmosis in patients is associated with parasite burden [11,27,28]. For instance, Stajner et al. [29] reported a fatal *T*. *gondii* reactivation in a human stem cell transplant recipient with underlying immunological deficiency, who had extremely high parasite burden in blood and BAL fluid. In the case of favorable outcome of reactivation in a heart transplant patient, a much low parasite load was observed [30].

Previously, the dopamine antagonists haloperidol and GBR 12909 were found to prevent the behavioural alterations in *T*. *gondii*-infected rats [31,32], suggesting dopamine has a role in *T*. *gondii*-induced behavioral changes. In the present study, we found that mice with high MAG1 antibody level displayed a striking behavioral deficit in amphetamine-trigged locomotor response. Our results are in agreement and extend prior studies by suggesting chronic *T*. *gondii* infection can trigger abnormal response to dopamine stimulant (amphetamine). Interestingly, this deficit was found not only associated with MAG1 antibody level, but also associated with IL-1β level. Amphetamine is thought to exert its stimulant effect by elevating synaptic concentrations of dopamine in brain reward areas. However, this deficit does not seem to be due to changes in striatal levels of dopamine, serotonin, and their metabolites, since no change was found in the striatum of mice with high MAG1 antibody level during chronic infection. This result is consistent with several reports describing no changes in monoamines and their metabolites during chronic infection [33,34]. In order to elucidate the mechanisms behind this deficit, dopamine transmission-related pathways might be considered in future studies.

Cognitive deficit has been reported in mice after infection with *T*. *gondii* [35,36]. In agreement with previous finding, there seems to be a general cognitive deficit in recognition memory in all *T*. *gondii* IgG seropositive mice (groups of MAG1 high, MAG1 low and without MAG1), although the differences did not reach significance in mice with MAG1 low and without MAG1 groups. Moreover, there was no correlation between cognitive impairment and MAG1 antibody levels. Our results suggested that immune response, not the presence of brain cysts, might contribute to the cognitive deficit. Although no correlation was found between cytokines and this deficit, this may depend on the cytokines involved. Interestingly, cognitive deficit was found at long time retention (24 h), but not at short time retention (1 h). This finding is in agreement with observation made by Gulinello et al. [37], who reported normal cognitive function after a 45 min delay in the object recognition assay.

Another possible reason for cognitive deficit in *T*. *gondii* IgG seropositive mice might include miR-132 dysregulation during infection. Similar to our previous result [24], we found miR-132 was downregulated during chronic infection in mice with high MAG1 antibody level. However, miR-132 was found to be upregulated during acute infection regardless of the parasite genotype, a phenomenon related to its effects on infection and inflammation [19]. It is worth noting that transgenic mice overexpressing miR-132 exhibited increased neuronal spine density but impaired novel object recognition memory [38,39]. Given miR-132 was upregulated before entering the chronic phase, this might be a potential cause for the observed deficit. Lately, miR-132 has been demonstrated to affect multiple neuronal functions and its dysregulation is linked to several neurological disorders [25]. Thus, miR-132 dysregulation provides a possible mechanism for changes in behavior and warrant further investigation.

In mice with high MAG1 antibody level, the expression of 3 proinflammatory cytokines (IL-1α, IL-12p70, and TNF-α) was decreased and expression of IL-12p40 and CCL5 was increased. The suppression of 3 proinflammatory cytokines indicated that the host did not mount a strong response directed at alerting and activating the immune system to react to the infection. These observations are in agreement with findings in humans. Yamamoto et al. [40] observed that asymptomatic persons had higher levels of IL-12 in response to *T*. *gondii* antigens than patients with ocular lesions. De-la-Torre et al. [41] reported that Colombian patients with severe ocular toxoplasmosis displayed a suppressive immune response, although the specific cytokines differ from our study. However, ongoing brain inflammation seems to be suggested, as evidenced by increased expression of markers associated with protective immunity. For example, as a well-established chemoattractant for T cells, the increased level of CCL5 could represent a potential mechanism that mediates CNS inflammation.

Here we reported that several behavioral abnormalities occurred in the mice with high MAG1 antibody level. Given this group of mice exhibited lower weight, this might be a confounding factor that contributes to the differences in behavior. It is possible that decreased activity in the open field may be due to lower weight, as sick mice do not move as much. However, the deficit seen in novel object recognition is less likely due to lower weight considering the deficit wasn't observed at 1 hr retention, but was seen at 24 hrs retention (presumably the weights were still lower). Similarly, the failed response to amphetamine could not simply be ascribed to the mice being lower weight, because it looks like all groups decreased their activity after the saline administration and are about the same before amphetamine treatment. Certainly, more experiments are necessary to confirm that this difference in response is specific to the amphetamine or dopamine pathway.

In conclusion, we were able to establish a model of chronic infection with virulent type 1 strain and to determine correlates of disease pathogenesis. Although it is known that parasite burden is associated with the degree of symptomatology in human toxoplasmosis, the present results have three major findings. First, MAG1 antibody level has predictive value for brain cyst burden and for changes in behavior or other pathology in chronically infected mice. Second, chronic *T*. *gondii* infection could trigger abnormal response to dopamine stimulant (amphetamine). Finally, our study suggested that some behaviors are associated with *T*. *gondii* itself (latent infection), whereas other behaviors do not require the presence of brain cysts (may be related to acute infection). This model may be useful in the performance of translational studies relating to human toxoplasmosis. These variable phenotypes displayed in this mouse model may reflect the diversity of human responses to *T*. *gondii* and lead to an increased understanding of mechanisms underlying pathogenic changes following infection.

## References

[1] Su C, Khan A, Zhou P, Majumdar D, Ajzenberg D, et al (2012) Globally diverse Toxoplasma gondii isolates comprise six major clades originating from a small number of distinct ancestral lineages. Proc Natl Acad Sci U S A 109: 5844–5849. 10.1073/pnas.1203190109 22431627 PMC3326454

[2] Howe DK, Sibley LD (1995) Toxoplasma gondii comprises three clonal lineages: correlation of parasite genotype with human disease. J Infect Dis 172: 1561–1566. 7594717 10.1093/infdis/172.6.1561

[3] Melo MB, Jensen KD, Saeij JP (2011) Toxoplasma gondii effectors are master regulators of the inflammatory response. Trends Parasitol 27: 487–495. 10.1016/j.pt.2011.08.001 21893432 PMC3200456

[4] Xiao J, Yolken RH (2015) Strain hypothesis of Toxoplasma gondii infection on the outcome of human diseases. Acta Physiol (Oxf) 213: 828–845. 25600911 10.1111/apha.12458 PMC4361247

[5] Khan A, Su C, German M, Storch GA, Clifford DB, et al (2005) Genotyping of Toxoplasma gondii strains from immunocompromised patients reveals high prevalence of type I strains. J Clin Microbiol 43: 5881–5887. 16333071 10.1128/JCM.43.12.5881-5887.2005 PMC1317192

[6] Ferreira IM, Vidal JE, Costa-Silva TA, Meira CS, Hiramoto RM, et al (2008) Toxoplasma gondii: genotyping of strains from Brazilian AIDS patients with cerebral toxoplasmosis by multilocus PCR-RFLP markers. Exp Parasitol 118: 221–227. 17950282 10.1016/j.exppara.2007.08.006

[7] Grigg ME, Ganatra J, Boothroyd JC, Margolis TP (2001) Unusual abundance of atypical strains associated with human ocular toxoplasmosis. J Infect Dis 184: 633–639. 11474426 10.1086/322800

[8] Xiao J, Buka SL, Cannon TD, Suzuki Y, Viscidi RP, et al (2009) Serological pattern consistent with infection with type I Toxoplasma gondii in mothers and risk of psychosis among adult offspring. Microbes Infect 11: 1011–1018. 10.1016/j.micinf.2009.07.007 19638313

[9] McLeod R, Boyer KM, Lee D, Mui E, Wroblewski K, et al (2012) Prematurity and severity are associated with Toxoplasma gondii alleles (NCCCTS, 1981–2009). Clin Infect Dis 54: 1595–1605. 10.1093/cid/cis258 22499837 PMC3348955

[10] Suzuki Y, Wong SY, Grumet FC, Fessel J, Montoya JG, et al (1996) Evidence for genetic regulation of susceptibility to toxoplasmic encephalitis in AIDS patients. J Infect Dis 173: 265–268. 8537674 10.1093/infdis/173.1.265

[11] Romand S, Chosson M, Franck J, Wallon M, Kieffer F, et al (2004) Usefulness of quantitative polymerase chain reaction in amniotic fluid as early prognostic marker of fetal infection with Toxoplasma gondii. Am J Obstet Gynecol 190: 797–802. 15042017 10.1016/j.ajog.2003.09.039

[12] Carme B, Demar M, Ajzenberg D, Darde ML (2009) Severe acquired toxoplasmosis caused by wild cycle of Toxoplasma gondii, French Guiana. Emerg Infect Dis 15: 656–658. 10.3201/eid1504.081306 19331765 PMC2671434

[13] Xiao J, Viscidi RP, Kannan G, Pletnikov MV, Li Y, et al (2013) The Toxoplasma MAG1 peptides induce sex-based humoral immune response in mice and distinguish active from chronic human infection. Microbes Infect 15: 74–83. 10.1016/j.micinf.2012.10.016 23142034 PMC4005331

[14] Hermes G, Ajioka JW, Kelly KA, Mui E, Roberts F, et al (2008) Neurological and behavioral abnormalities, ventricular dilatation, altered cellular functions, inflammation, and neuronal injury in brains of mice due to common, persistent, parasitic infection. J Neuroinflammation 5: 48 10.1186/1742-2094-5-48 18947414 PMC2588578

[15] Xiao J, Kannan G, Jones-Brando L, Brannock C, Krasnova IN, et al (2012) Sex-specific changes in gene expression and behavior induced by chronic Toxoplasma infection in mice. Neuroscience 206: 39–48. 10.1016/j.neuroscience.2011.12.051 22240252

[16] Kannan G, Moldovan K, Xiao JC, Yolken RH, Jones-Brando L, et al (2010) Toxoplasma gondii strain-dependent effects on mouse behaviour. Folia Parasitol (Praha) 57: 151–155. 20608478 10.14411/fp.2010.019

[17] Melnikova T, Savonenko A, Wang Q, Liang X, Hand T, et al (2006) Cycloxygenase-2 activity promotes cognitive deficits but not increased amyloid burden in a model of Alzheimer's disease in a sex-dimorphic pattern. Neuroscience 141: 1149–1162. 16753269 10.1016/j.neuroscience.2006.05.001

[18] Yolken RH, Jones-Brando L, Dunigan DD, Kannan G, Dickerson F, et al (2014) Chlorovirus ATCV-1 is part of the human oropharyngeal virome and is associated with changes in cognitive functions in humans and mice. Proc Natl Acad Sci U S A 111: 16106–16111. 10.1073/pnas.1418895111 25349393 PMC4234575

[19] Xiao J, Li Y, Prandovszky E, Karuppagounder SS, Talbot CC Jr., et al (2014) MicroRNA-132 dysregulation in Toxoplasma gondii infection has implications for dopamine signaling pathway. Neuroscience 268: 128–138. 10.1016/j.neuroscience.2014.03.015 24657774 PMC4085776

[20] Ueno A, Dautu G, Munyaka B, Carmen G, Kobayashi Y, et al (2009) Toxoplasma gondii: Identification and characterization of bradyzoite-specific deoxyribose phosphate aldolase-like gene (TgDPA). Exp Parasitol 121: 55–63. 10.1016/j.exppara.2008.09.018 18950626

[21] Couper KN, Roberts CW, Brombacher F, Alexander J, Johnson LL (2005) Toxoplasma gondii-specific immunoglobulin M limits parasite dissemination by preventing host cell invasion. Infect Immun 73: 8060–8068. 16299300 10.1128/IAI.73.12.8060-8068.2005 PMC1307022

[22] Gaskell EA, Smith JE, Pinney JW, Westhead DR, McConkey GA (2009) A unique dual activity amino acid hydroxylase in Toxoplasma gondii. PLoS One 4: e4801 10.1371/journal.pone.0004801 19277211 PMC2653193

[23] McConkey GA, Martin HL, Bristow GC, Webster JP (2013) Toxoplasma gondii infection and behaviour—location, location, location? J Exp Biol 216: 113–119. 10.1242/jeb.074153 23225873 PMC3515035

[24] Li YE, Kannan G, Pletnikov MV, Yolken RH, Xiao J (2015) Chronic infection of Toxoplasma gondii downregulates miR-132 expression in multiple brain regions in a sex-dependent manner. Parasitology 142: 623–632. 10.1017/S003118201400167X 25351997 PMC4428143

[25] Wanet A, Tacheny A, Arnould T, Renard P (2012) miR-212/132 expression and functions: within and beyond the neuronal compartment. Nucleic Acids Res 40: 4742–4753. 10.1093/nar/gks151 22362752 PMC3367188

[26] Ferguson DJ, Parmley SF (2002) Toxoplasma gondii MAG1 protein expression. Trends Parasitol 18: 482 12473362 10.1016/s1471-4922(02)02349-8

[27] Costa JG, Carneiro AC, Tavares AT, Andrade GM, Vasconcelos-Santos DV, et al (2013) Real-time PCR as a prognostic tool for human congenital toxoplasmosis. J Clin Microbiol 51: 2766–2768. 10.1128/JCM.00982-13 23761154 PMC3719656

[28] Shobab L, Pleyer U, Johnsen J, Metzner S, James ER, et al (2013) Toxoplasma serotype is associated with development of ocular toxoplasmosis. J Infect Dis 208: 1520–1528. 10.1093/infdis/jit313 23878321 PMC3789564

[29] Stajner T, Vasiljevic Z, Vujic D, Markovic M, Ristic G, et al (2013) Atypical strain of Toxoplasma gondii causing fatal reactivation after hematopoietic stem cell transplantion in a patient with an underlying immunological deficiency. J Clin Microbiol 51: 2686–2690. 10.1128/JCM.01077-13 23761151 PMC3719651

[30] Patrat-Delon S, Gangneux JP, Lavoue S, Lelong B, Guiguen C, et al (2010) Correlation of parasite load determined by quantitative PCR to clinical outcome in a heart transplant patient with disseminated toxoplasmosis. J Clin Microbiol 48: 2541–2545. 10.1128/JCM.00252-10 20463167 PMC2897503

[31] Webster JP, Lamberton PH, Donnelly CA, Torrey EF (2006) Parasites as causative agents of human affective disorders? The impact of anti-psychotic, mood-stabilizer and anti-parasite medication on Toxoplasma gondii's ability to alter host behaviour. Proc Biol Sci 273: 1023–1030. 16627289 10.1098/rspb.2005.3413 PMC1560245

[32] Skallova A, Kodym P, Frynta D, Flegr J (2006) The role of dopamine in Toxoplasma-induced behavioural alterations in mice: an ethological and ethopharmacological study. Parasitology 133: 525–535. 16882355 10.1017/S0031182006000886

[33] Gatkowska J, Wieczorek M, Dziadek B, Dzitko K, Dlugonska H (2013) Sex-dependent neurotransmitter level changes in brains of Toxoplasma gondii infected mice. Exp Parasitol 133: 1–7. 10.1016/j.exppara.2012.10.005 23098668

[34] Wang ZT, Harmon S, O'Malley KL, Sibley LD (2015) Reassessment of the role of aromatic amino acid hydroxylases and the effect of infection by Toxoplasma gondii on host dopamine. Infect Immun 83: 1039–1047. 10.1128/IAI.02465-14 25547791 PMC4333481

[35] Witting PA (1979) Learning capacity and memory of normal and Toxoplasma-infected laboratory rats and mice. Z Parasitenkd 61: 29–51. 543216 10.1007/BF00927085

[36] Hodkova H, Kolbekova P, Skallova A, Lindova J, Flegr J (2007) Higher perceived dominance in Toxoplasma infected men—a new evidence for role of increased level of testosterone in toxoplasmosis-associated changes in human behavior. Neuro Endocrinol Lett 28: 110–114. 17435678

[37] Gulinello M, Acquarone M, Kim JH, Spray DC, Barbosa HS, et al (2010) Acquired infection with Toxoplasma gondii in adult mice results in sensorimotor deficits but normal cognitive behavior despite widespread brain pathology. Microbes Infect 12: 528–537. 10.1016/j.micinf.2010.03.009 20348009 PMC2891993

[38] Hansen KF, Sakamoto K, Wayman GA, Impey S, Obrietan K (2010) Transgenic miR132 alters neuronal spine density and impairs novel object recognition memory. PLoS One 5: e15497 10.1371/journal.pone.0015497 21124738 PMC2993964

[39] Hansen KF, Karelina K, Sakamoto K, Wayman GA, Impey S, et al (2013) miRNA-132: a dynamic regulator of cognitive capacity. Brain Struct Funct 218: 817–831. 10.1007/s00429-012-0431-4 22706759 PMC3508255

[40] Yamamoto JH, Vallochi AL, Silveira C, Filho JK, Nussenblatt RB, et al (2000) Discrimination between patients with acquired toxoplasmosis and congenital toxoplasmosis on the basis of the immune response to parasite antigens. J Infect Dis 181: 2018–2022. 10837184 10.1086/315494

[41] de-la-Torre A, Sauer A, Pfaff AW, Bourcier T, Brunet J, et al (2013) Severe South American ocular toxoplasmosis is associated with decreased Ifn-gamma/Il-17a and increased Il-6/Il-13 intraocular levels. PLoS Negl Trop Dis 7: e2541 10.1371/journal.pntd.0002541 24278490 PMC3837637

